# Applying a data-driven approach to quantify EEG maturational deviations in preterms with normal and abnormal neurodevelopmental outcomes

**DOI:** 10.1038/s41598-020-64211-0

**Published:** 2020-04-29

**Authors:** Kirubin Pillay, Anneleen Dereymaeker, Katrien Jansen, Gunnar Naulaers, Maarten De Vos

**Affiliations:** 1 Institute of Biomedical Engineering (IBME), Department of Engineering Science, University of Oxford, Oxford, United Kingdom; 2 Department of Paediatrics, John Radcliffe Hospital, University of Oxford, Oxford, United Kingdom; 3 Department of Development and Regeneration, University Hospitals Leuven, Neonatal Intensive Care Unit, KU Leuven (University of Leuven), Leuven, Belgium; 4 Department of Development and Regeneration, University Hospitals Leuven, Child Neurology, University of Leuven (KU Leuven), Leuven, Belgium

**Keywords:** Neonatal brain damage, Preterm birth, Quality of life, Biomedical engineering, Scientific data

## Abstract

Premature babies are subjected to environmental stresses that can affect brain maturation and cause abnormal neurodevelopmental outcome later in life. Better understanding this link is crucial to developing a clinical tool for early outcome estimation. We defined maturational trajectories between the Electroencephalography (EEG)-derived ‘brain-age’ and postmenstrual age (the age since the last menstrual cycle of the mother) from longitudinal recordings during the baby’s stay in the Neonatal Intensive Care Unit. Data consisted of 224 recordings (65 patients) separated for normal and abnormal outcome at 9–24 months follow-up. Trajectory deviations were compared between outcome groups using the root mean squared error (*RMSE*) and maximum trajectory deviation (*δ*^*max*^). 113 features were extracted (per sleep state) to train a data-driven model that estimates brain-age, with the most prominent features identified as potential maturational and outcome-sensitive biomarkers. *RMSE* and *δ*^*max*^ showed significant differences between outcome groups (cluster-based permutation test, p < 0.05). *RMSE* had a median (IQR) of 0.75 (0.60–1.35) weeks for normal outcome and 1.35 (1.15–1.55) for abnormal outcome, while *δ*^*max*^ had a median of 0.90 (0.70–1.70) and 1.90 (1.20–2.90) weeks, respectively. Abnormal outcome trajectories were associated with clinically defined dysmature and disorganised EEG patterns, cementing the link between early maturational trajectories and neurodevelopmental outcome.

## Introduction

Preterm birth gives children an increased risk of neurodevelopmental impairments resulting in motor, cognitive, functional or emotional disabilities. Susceptibility for, and the severity of, these complications rise with decreasing gestational age (GA = the age at birth since the last menstrual cycle of the mother) and with an acquired injury to the developing brain^1^. Increased survival, especially from the most extreme preterm infants, requires efforts to improve the early prediction of abnormal neurodevelopmental outcome. An enhanced understanding of key maturational biomarkers linked to long-term outcome during the period in the Neonatal Intensive Care Unit (NICU) would better ensure optimal brain development and facilitate an automated assessment of outcome^2,3^.

Implementation of continuous background Electroencephalogram (EEG) monitoring aims to observe the state of brain development, in order to recognize early markers of abnormal brain maturation and provide opportunities for early intervention^4^. As preterm babies spend the majority of their time in sleep, previous clinical research has suggested the use of sleep ontogenesis (the changing nature of sleep cycles with age) as a means to quantify brain maturation and identify abnormal deviations^5,6^. EEG provides a means to distinctly quantify sleep ontogeny, clearly capturing these changes as the baby matures. Below 28 weeks postmenstrual age (PMA = GA plus the time elapsed after birth), the baby exhibits very crude sleep behaviours but beyond this age, the development of deeper neuronal connections results in the clear differentiation between periods of wakefulness, Active Sleep (AS, or Rapid Eye Movement (REM) sleep), and Quiet Sleep (QS, or non-REM sleep)^7^.

Brain activity reflects a complex interaction between different homeostatic mechanisms that regulate cerebral haemodynamics and metabolism^8,9^. While changes in cerebral oxygenation, carbon dioxide levels, blood pressure and drugs mostly trigger transient (acute) alterations in brain activity^10–13^, previous research has also highlighted two distinct patterns of more permanent (chronic) EEG abnormalities, which can be best observed in sequential (longitudinal) EEG recordings of growing, stable babies.

During normal brain development, it is assumed that EEG sleep states will exhibit patterns that reflect the actual age, i.e. that the baby’s ‘brain-age’ is matched. However, chronic abnormal EEG patterns may instead exhibit dysmature behaviours resulting in delayed or accelerated sleep development and, consequently, EEG brain-ages that under- or over-estimate the true age^14,15^. Disorganized patterns are also observed, producing a deformed, abnormal sharp transient activity compared to normal background EEG^16^. In addition, background EEG also can also show a combination of both dysmature and disorganised patterns and both have been previously identified visually for clinical diagnoses and prognoses^14,15,17,18^.

A number of studies have visually graded serial EEGs as dysmature and/or disorganised with proven correlations to neurodevelopmental outcome. However, such grading is prone to interrater variability and requires a visual assessment of each recording^19^ so does not lend itself to an automated solution. Consequently, a number of background EEG features have been derived as objective biomarkers to automatically quantify the maturing EEG sleep patterns with age^2,19–25^. Existing studies that attempt to correlate background EEG measures with neurodevelopmental outcome typically monitor babies up to 72 hours–6 weeks^19,25,26^, while longer-range studies are limited to 33 weeks PMA^27^ or still rely on the visual grading of the background EEG^2,27,28^. The timing of identifying maturational deviations is crucial and varies with PMA, yet there remains no study that links brain maturation with neurodevelopmental outcome over the full preterm to term age, utilising objective biomarkers to prevent the need for individual visual EEG grading.

With the lack of such a ground truth at the time of recording, it is unknown as to which recording(s) may deviate if the patient exhibits abnormal neurodevelopmental outcome. Some recordings may still exhibit normal maturational patterns, hindering standard analytical approaches such as directly correlating a recording’s features with outcome, or performing outcome classification at the recording level. Instead, assessing how each patient’s recordings change with PMA by forming a developmental trajectory would be more appropriate.

Previous engineering studies have combined EEG features to successfully derive and validate brain-age prediction models from normal outcome data, also known as the Expected Maturational Age (EMA)^25,29–31^. Summarizing each patient’s recordings with such models and forming a brain-age trajectory^30^ provides an intuitive and interpretable measure of brain maturation.

The aim of this study is thus two-fold. First, we derive brain-age prediction models from a large set of EEG features, forming trajectories and assessing differences in defined trajectory summary metrics with relation to neurodevelopmental outcome. Second, we assess the contributions of individual features in these models to identify objective biomarkers of maturation and outcome.

## Methods

### Datasets and outcome assessment

Data was recorded from the NICU of the University Hospitals, Leuven (Belgium) in accordance with the relevant guidelines and regulations and approved by the ethics committee of the University Hospitals, Leuven. All patients were recruited after informed consent from the parents. For each patient, between one and five EEGs were recorded at different PMAs over the period the patient remained in the NICU. All EEGs were measured using the standard 10–20 electrode system with nine channels (Fp1, Fp2, C3, C4, T3, T4, O1, O2 and reference Cz) at a sampling frequency of 250 Hz.

Two separate cohorts of patients were recruited, resulting in two separate EEG datasets referred to as ${\mathcal{D}}1$ and ${\mathcal{D}}2$ in this study, respectively. ${\mathcal{D}}1$ consisted of 89 EEG recordings from 26 patients with PMA range 27.3–42.0 weeks (median recording duration (IQR): 4 h 23 m (3 h 53 m-5 h 46 m), median number of recordings per patient: 3 (3–4)) and were recruited in the period February 2013 - September 2014. These were preselected based on a normal neurodevelopmental outcome at nine and 24 months of corrected age, clinically defined according to *Pillay et al*.^32^ and *Dereymaeker et al*.^33^ and based on the Bayley Scores of Infant mental and motor Development II (BSID-II).

${\mathcal{D}}2$ consisted of 135 recordings from 39 patients with PMA range of 26.4–42.0 weeks (median recording duration: 4 h 04 m (3 h 25 m-5 h 16 m), median number of recordings per patient: 3 (2–4)) and had BSID-II scores at nine months of corrected age. ${\mathcal{D}}2$ was recruited in the period July 2013 – December 2015. This dataset further included both normal and abnormal outcome patients organised into groups based on their severity of abnormality:

1. Normal outcome: 68 recordings (21 patients) selected using the same criteria as ${\mathcal{D}}1$.
2. Mild abnormal outcome: 33 recordings (nine patients) with BSID-II scores (motor or mental) range 71–84.
3. Severe abnormal outcome: 20 recordings (six patients) with a BSID-II score (motor or mental) ≤70 and/or the presence of cerebral palsy.
4. Lost-to-follow-up/death: 14 recordings (three patients) who died before follow-up at nine months.

A combined clinical summary of both datasets, sorted by outcome group, is presented in Table 1. Appropriate statistical tests were performed to check if there were any unexpected significant differences in the measures/treatments between outcome groups.

**Table 1:** Combined demographic summary of ${\mathcal{D}}1$ and ${\mathcal{D}}2$. Normal outcome recordings from both datasets are combined in this table.

	Normal	Mild abnormal	Severe abnormal	Death	group *p*-value
Patient number, n (%)	47 (72)	9 (14)	6 (9)	3 (5)	
Gestational age in weeks, mean (SD)	28.7 (2.2)	29.2 (1.7)	28.8 (2.9)	26.0 (1.2)	0.201
range	24.6–32	26.3–31.3	25–31.7	24.9–27.3	
Sex male, %	51%	100%	50%	67%	0.012
**Twin**					
DCDA	18	0	0	1	0.24
MCDA	4	3	1	0	
Birth weight in g, mean (SD)	1266 (415)	1256 (354)	1152 (529)	796 (72)	0.278
range	540–2540	760–1700	485–1730	750–880	
SGA^a^, n (%)	3 (6)	1 (11)	2 (33)	0	0.285
Laser therapy for Retinopathy of Prematurity, n (%)	6 (13)	1 (11)	2 (33)	2 (67)	0.370
Bronchopulmonary Dysplasia^b^, n (%)	7 (15)	2 (22)	3 (50)	2 (67)	0.020
Necrotizing enterocolitis ≥ Bell stage 2, n (%)	2 (4)	0	0	1 (33)	0.824
Single Intestinal Perforation	2 (4)	0	0	0	
Sepsis^c^, n (%)	23 (49)	5 (56)	2 (33)	3 (100)	0.175
Patent ductus arteriosus treatment, n (%)	12 (26)	2 (22)	2 (33)	2 (67)	0.511
**Ventilatory support*, days***					
Mechanical ventilation, mean (range)	4.8 (0–35)	3.7 (0–17)	10.5 (0–34)	20.3 (7–32)	0.285
Non-invasive ventilation, mean (range)	18.0 (0–63)	23.0 (1–55)	31.0 (3–87)	45.0 (27–71)	0.095
**Cranial Ultrasound**^**d**^ **(%)**					
Normal	32	7	1	1	0.013
Mild brain lesion (grade I-II)	12	1	2	0	
Severe brain lesion	3	1	1	2	
Other	0	0	2	0	
**Bayley Scale of Infant**					
**Development-II**					
Mental mean (SD)	105 (11)	84 (8)	66 (12)	/	<0.001
Motor mean (SD)	106 (14)	94 (15)	62 (11)	/	<0.001

Values are expressed as n (%), mean and standard deviation (SD) or range. Tested with ANOVA and Kruskal-Wallis test for continuous data or χ^2^ Likehood ratio test for categorical data. Group 1 (normal): mental (MDI), motor (PDI) ≥ 85; Group 2 (mild abnormal): MDI or PDI 71–84; Group 3 (severe abnormal): MDI or PDI ≤ 70, cerebral palsy. p-values are listed for groups 1–2–3–4.

^a^Small for gestational age (SGA) defined as birth weight <2 SDs below the mean for an infant’s gestational age.

^b^Bronchopulmonary Dysplasia defined as requiring supplemental oxygen/ventilatory therapy at 36 weeks PMA. ^c^Sepsis: positive blood culture or highly suspected with antibiotic treatment >72 h.

^d^Cranial ultrasound: Mild brain lesion = IVH grade I–II and/or PVL grade I; severe brain lesion = IVH grade III and/or PVH and/or PVL grade II–III. Other: isolated corpus callosum agenesis.

Finally, for this study, mild abnormal, severe abnormal, and death were combined into a single abnormal outcome group.

### EEG pre-processing

Each EEG recording was bandpass filtered in the range 0.5–40 Hz and visually labelled for periods of artefacts (such as electrode drop-offs and movement) by a trained clinician (AD).

Accounting for differences across sleep state was an important consideration^34^, with the previously developed CLASS algorithm for the robust detection of QS (see *Dereymaeker et al*.^33^) applied to each recording to obtain QS and non-QS (comprising both AS and wakefulness) periods while excluding periods that were also labelled as artefact. To additionally compare the effects of separating for sleep state, clean, non-sleep state specific two-hour periods of EEG were also extracted (incorporating at least one full sleep cycle).

Within each QS, non-QS, and full-cycle period, 113 different features were extracted from each channel in 30 second epochs. The majority of this feature set have also been used and defined in a previous study on automated sleep staging in term-age babies (see *Pillay et al*.^32^), and is summarised in Table 2. Further technical descriptions of these features are detailed in Appendix A.1.

**Table 2:** See also *Pillay et al*.^32^ and Appendix A.1.

Feature category	Feature description
**TIME DOMAIN**	
	SD, mean absolute amplitudes, Max. Absolute amplitude, and sum of first and second derivatives, Max-min difference of amplitudes, Skewness and Kurtosis, Hjorth activity, complexity, mobility, ApEn, SampEn, and MSE (scales 1-10), SD of MSE values, Area under multiscale curve, Average slope of multiscale curve (scales 1-5), Average slope of multiscale curve (scales 6-20), Max MSE value, Katz and Higuchi Fractal Dimensions (kmax=20,40,60), Zero crossing rate SD, Coastline distance, Mean and SD TKEO values, 90% peak value, width, prominence, Line Length suppression value (supp), Line Length Burst% Mean, median, LM (5^th^ percentile), HM (95^th^ percentile), SD, IQR, and skewness of rEEG. SD and mean absolute amplitudes in delta, theta, alpha, beta frequency bands, Hilbert median envelope amplitude, delta, theta, Hilbert median instantaneous frequency, delta, theta bands.
***Wavelet decomposition***	Mean absolute value and SD of amplitudes in D3, D4, D5, A5 bands, Ratio of absolute mean values in adjacent sub bands, Sum of squared coefficients in D3, D4, D5, A5 bands.
***EMD***	Variation coefficients of IMF1-IMF6, Fluctuation index of IMF1-IMF6, Mean absolute ratio of each pair of successive IMFs, Hilbert median instantaneous frequency, IMF1-IMF6.
**FREQUENCY DOMAIN**	
***Fourier-transform***	Full band power (delta-beta range), Mean frequency, Spectral roll-off SD, centroid SD, flux SD, Power spectral entropy, 90% spectral edge frequency, Relative band power in delta, theta, alpha, beta bands, Mean frequency in delta, theta, alpha, beta bands, EEG spectral beta/alpha ratio,

Features calculated from each 30 second epoch, including time domain and frequency domain measures.

SD: Standard Deviation, IQR: Interquartile Range, ApEn: Approximate Entropy, SampEn: Sample Entropy, MSE: Multiscale Entropy, EMD: Empirical Mode Decomposition, IMF: Intrinsic Mode Function, TKEO: Teager-Kaiser Energy Operator.

delta: 0.5–3 Hz, theta: 3–8 Hz, alpha: 8–12 Hz, beta: 12–30 Hz.

Features were selected across a wide range of preterm, infant and adult EEG studies as part of a data-driven approach, which utilizes the data and subsequent analyses to identify important features rather than pre-selecting them based on prior clinical knowledge. Medians were taken across each channel (unless the feature utilised all channels) and epoch^30^ resulting in a total of 339 features (113 from QS, 113 from non-QS and 113 from the full-cycle periods) per recording.

Obtaining median values across the full recording ensured a robust approach for estimating the most representative features for the given sleep state and recording, reducing the risk of additional variability that may be brought about by any residual or missed short-duration artefacts (due to muscle twitch^33^ etc.).

**Figure 1:**
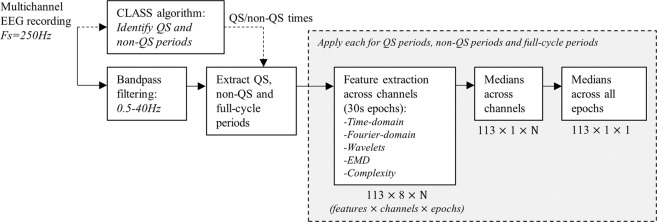
Pre-processing procedure for extracting a representative set of features for each recording from QS, non-QS and full-cycle EEG periods. Dimensions of the resulting output is provided below each stage, where applicable.

A summary of this pre-processing procedure is presented in Fig. 1.

### Brain-age prediction and deriving trajectories

With a 24-month normal neurodevelopment at follow-up, ${\mathcal{D}}1$ provided the most reliable estimate of a normal developing EEG and has been used extensively in previous studies on preterm brain maturation and sleep staging^23,33,35–38^. Consequently, PMA was considered a reasonable estimate of EEG brain-age in this dataset^30^ and made it ideal as the training set for the brain-age prediction model. While ${\mathcal{D}}2$ also included normal outcome data, this was assessed at only nine months follow-up so this could not be combined with ${\mathcal{D}}1$ for training. Consequently, all of ${\mathcal{D}}2$ (normal and abnormal outcome data) was kept separate from ${\mathcal{D}}1$ throughout the analyses.

To perform brain-age prediction, we used a Random Forest (RF) regression model.

#### The RF regression model

The RF is ideally suited to data where the number of features is greater than the number of recordings, does not require prior standardization, and intrinsically selects those features that are most predictive without the need for additional feature selection. Briefly, an RF consists of a series of decision trees. Each tree is ‘grown’ by selecting a feature from the full feature set that best splits the training data, with the process then subsequently repeated on the split data and so on, until a stopping criterion is achieved. Note that the same feature can be selected multiple times in a single tree. Finally, a new test data point is then passed through this trained tree to generate a regression estimate.

In an RF, instead of choosing from all features for each split in the tree, a subset of ${N}^{f}$ features are selected at random each time. A total of ${N}^{trees}$ trees are trained, and the average obtained across the trees for the final estimate. Optimal feature choices at each split are assessed by maximising the *drop* in the Sum of Squared Error ($\Delta SSE$) between the estimates and true values. The overall $\Delta SSE$ for each feature (across all splits and trees) additionally provides a measure of the feature importance in the model.

#### Deriving brain-age trajectories

RF brain-age models were trained using the PMA as the training labels and combining the features from both QS and non-QS (226 features per recording). Each brain-age trajectory was then derived by plotting the patient’s brain age recording estimates against their PMA.

Typically, the choice of the RF parameter ${N}^{trees}$ is not very sensitive if set sufficiently high so we selected a value of 1500 throughout. Choosing ${N}^{f}$, however, affects the choice and variation of features used by the RF. A larger number of *N*^*f*^ reduces the *variability* of the features selected across trees, while smaller *N*^*f*^ results in a wider range of the feature set represented^39^. As the aim of identifying abnormal deviations linked to neurodevelopmental outcome may be strongly dependent on this choice, training and testing was repeated and presented for all values of *N*^*f*^ across the full range (i.e. *N*^*f*^ = 1 to *N*^*f*^ = 226).

Various studies have presented the effects of RF training at extreme values of *N*^*f*^, with differing conclusions^39–41^. Therefore, before comparing between the normal and abnormal outcomes, we performed a preliminary study to first assess the stability of the RF across the full *N*^*f*^ range using only the normal-outcome data at 24 months follow up (${\mathcal{D}}1$). ${\mathcal{D}}1$ was used for training and testing the models by Leave-One-Subject-Out Cross-Validation (LOSO CV). This would also importantly confirm if a strong correlation was observed between the brain-age estimates and PMA.

We then trained RF models on the entirety of the feature data for ${\mathcal{D}}1$ before testing this model on ${\mathcal{D}}2$ to derive the brain age trajectories for each outcome group (across the full *N*^*f*^ range). Although outcome was not explicitly used during training and testing, the deviation of these trajectories was hypothesized to increase in patients with abnormal outcome. From the trained models, the most prominently selected features were then identified.

As a supplementary analysis to confirm the benefits of combining both QS and non-QS features in the model, we also repeated the above analysis on various alternative models that were trained using features from a) only QS, b) only non-QS, and c) the full-cycle periods, for comparison.

### Trajectory summary metrics

With brain-age trajectories estimated for each patient, metrics were next derived to summarize them. When comparing trajectories between the normal and abnormal outcome groups, the precise nature of potential deviations was unknown. Therefore, three different metrics were chosen to capture different aspects of the deviation:

1. The trajectory’s Root Mean-Squared Error ($RMSE$) is a standard measure of the overall regression accuracy between brain-age and PMA and was calculated here across the recordings for each patient trajectory.
2. The adjusted correlation coefficient (*r*) was calculated to provide a measure of the trajectory correlation strength with PMA^30^. A linear mixed-effects model was fitted to the output, with brain-age as the response variable, PMA as the predictor variable and a random intercept to account for the repeated recordings in each trajectory. The square root of the adjusted R^2^ resulted in *r*. Extremities of 1 denotes a perfect linear correlation and 0 denotes no correlation.
3. The maximum deviation (*δ*^*max*^) measures the maximum deflection of each trajectory from the normal trend. A regression line was first fitted to the training output from ${\mathcal{D}}1$ to define the normal trend, and the absolute difference between each test recording and this fitted line was determined. The maximum absolute difference was then chosen to summarize each trajectory.

### Statistical tests

When comparing the values of the trajectory summary metrics between the normal and abnormal outcome groups across the full range of ${N}^{f}$, the Wilcoxon rank-sum test was calculated at each value of ${N}^{f}$ to determine statistically significant differences at p < 0.05. To correct for multiple comparisons, a cluster-based non-parametric permutation test was then applied by grouping significant values at adjacent ${N}^{f}$ to form clusters and using the total rank-sum values within each cluster as the cluster mass test statistic (see *Maris & Oostenveld (2007)*^42^). Statistically significant clusters were identified at p < 0.05 (one-tailed test).

To further assess if the features that proved prominent in the trained models could be considered as potential biomarkers for maturation and outcome, a univariate linear mixed-effects model was fitted for each identified feature, with fixed effects for the PMA and outcome and random intercepts to account for the repeated recordings. The p-values for the fixed effects were assessed for significance at p < 0.05.

## Results

### Preliminary assessment of model stability and brain-age correlation

**Figure 2:**
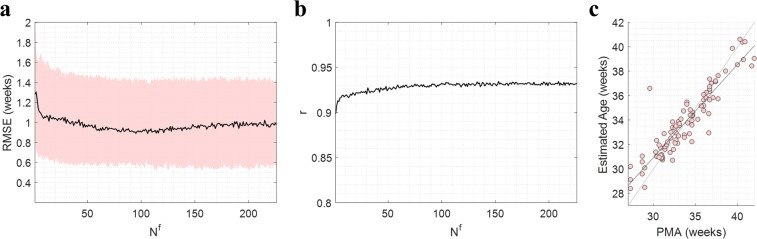
Assessing stability of the RF model and brain-age correlation with PMA over the full range of *N*^*f*^. Models are tested and trained on ${\mathcal{D}}1$ using Leave-One-Subject-Out cross-validation. (**a**) Results for the trajectory $RMSEs$. Black lines denote the median values and shaded regions denote the interquartile ranges. (**b**) Results for $r$. (**c**) Scatter plot of estimated brain-age against PMA at *N*^*f*^ = 226. Grey line denotes estimated age = PMA and black line denotes the regression line (used to calculate $r$).

Figure 2 shows the results of the preliminary study to assess RF stability and brain-age correlation with PMA (using combined QS and non-QS feature sets). The trajectory RMSEs and $r$ across ${N}^{f}$ are shown in Fig. 2a,b, respectively, on the left-out data after performing LOSO on ${\mathcal{D}}1$.

After an initial under-fitting up to *N*^*f*^ = 40, results showed an accurate and consistently stable model up to the maximum value of *N*^*f*^ = 226 with $RMSE$ (median (IQR)) in the range 0.90 (0.55–1.45) - 1.05 (0.60–1.50) weeks. Values of $r$ were also consistently stable in the range *N*^*f*^ = 40 to *N*^*f*^ = 226, remaining in the range 0.92–0.94. This confirmed that the model exhibited a stable behaviour up to the highest values of *N*^*f*^, with values of $r$ suggesting a strong correlation between the estimated brain-age and PMA throughout. Figure 2c shows a scatter plot of the estimated age against PMA at the maximum *N*^*f*^ = 226, further illustrating this strong positive correlation.

### Linking brain-age trajectories with neurodevelopmental outcome

**Figure 3:**
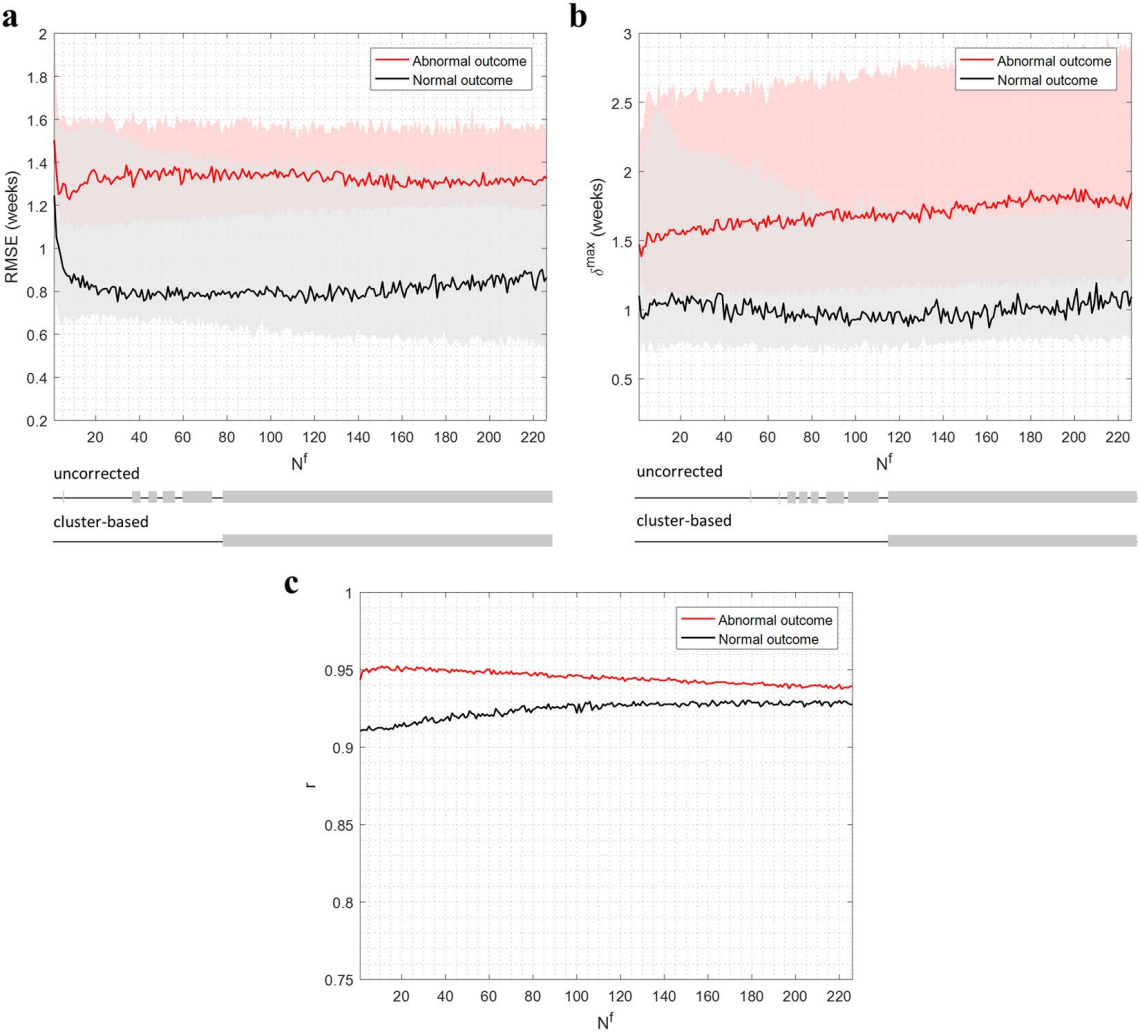
Overall brain-age trajectory performance on ${\mathcal{D}}2$ across *N*^*f*^. Results are separated for normal and abnormal neurodevelopmental outcome. Lines denote the medians and shaded regions denote the interquartile ranges. Below the plot are the results for both the uncorrected test for statistically significant differences between outcome groups (using the Wilcoxon rank-sum test), as well as the cluster-based non-parametric permutation test (p < 0.05) which corrects for multiple comparisons. (**a**) Results for the trajectory summary metric, $RMSE$. (**b**) Corresponding results for *δ*^*max*^. (**c**) Corresponding results for *r*. In this case, single values were calculated for each *N*^*f*^ so no statistical comparison was performed.

Figure 3 shows the overall test performance on ${\mathcal{D}}2$ after training on the entirety of ${\mathcal{D}}1$. The trajectory summary metrics are shown for all values of *N*^*f*^, and compared between the normal and abnormal outcome groups.

Figure 3a shows the results of $RMSE$. After initial underfitting of the models, results showed a clear, stable separability between the outcome groups in the range *N*^*f*^ = 20 to *N*^*f*^ = 226, with deviations in brain-age trajectories consistently greater in the abnormal outcome group. After performing the cluster-based non-parametric permutation test (at p < 0.05), a single significant cluster was found in the range *N*^*f*^ = 78 to *N*^*f*^ = 226 (p = 0.015). Within this cluster, the location of the largest median difference in $RMSE$ between the outcome groups had a median of 0.75 (0.60–1.35) weeks for the normal outcome group and 1.35 (1.15–1.55) for the abnormal outcome group. We also compared the abnormal subgroups of mild and severe outcome at the same location (ignoring the death subgroup due to limited patients) to assess if there were further differences. Mild abnormal outcome had a lower median $RMSE$ (1.19 (0.75–1.42)) than severe abnormal outcome (1.36 (1.27–2.48)) although this was not statistically significant.

Figure 3b shows similar results for *δ*^*max*^, with a significant cluster observed in the range *N*^*f*^ = 114 to *N*^*f*^ = 226 (p = 0.015) between normal and abnormal outcome. The location of largest difference within this cluster had a median *δ*^*max*^ of 0.90 (0.70–1.70) weeks for normal outcome and 1.90 (1.20–2.90) weeks for abnormal outcome. As with $RMSE$, a further comparison between the mild and severe abnormal subgroups revealed no statistically significant difference, though the mild abnormal subgroup had a lower median *δ*^*max*^ of 1.28 (1.10–2.25) than the severe abnormal subgroup of 1.80 (1.33–3.70).

Figure 3c compares the values of $r$ between the outcome groups. No cluster analysis was applied here as single values of $r$ were compared over *N*^*f*^. Values of $r$ remained very high at >0.9 across *N*^*f*^, with the difference between the outcome groups dropping to 0.01. These minor differences at higher *N*^*f*^ (and the counter-intuitively higher correlation in abnormal outcome than normal outcome) suggested that these were only due to small discrepancies between the data distribution in the outcome groups rather than a meaningful difference.

**Figure 4:**
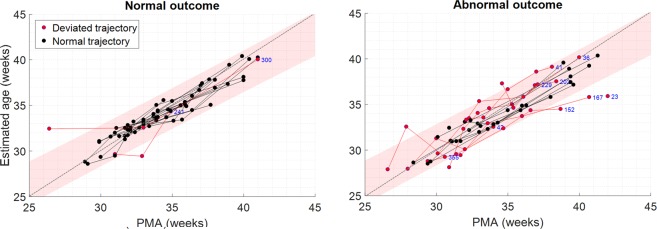
Illustration of the trajectory plots of ${\mathcal{D}}1$ for *N*^*f*^ = 226, separated by outcome group. The more deviant trajectories are highlighted by fitting a 95% prediction interval threshold to the normal-outcome data (left plot). The 95% prediction interval is shaded in each plot, with the trajectories highlighted in red when at least one recording exceeded this threshold. The anonymous patient ID is also provided for these cases (in blue). Grey dotted lines show the line for brain-age = PMA.

Achieving similarly high results for $r$ between groups, while still observing large differences between RMSE and *δ*^*max*^, suggested that the trajectories could be largely driven by single-recording deviations. To confirm this, we plotted the trajectories in Fig. 4 (separated for outcome) at *N*^*f*^ = 226 as all significant clusters for the trajectory metrics incorporated the maximum value of *N*^*f*^.

From Fig. 4, comparatively more deviant trajectories were emphasized by fitting a regression on the normal outcome trajectories (left) and calculating the 95% prediction interval as a simple threshold. Any patient trajectory where at least one recording exceeded this threshold was then highlighted in red. In general, this illustrated that while a few trajectories showed a systematic deviation, the majority were largely influenced by the effects of single recordings. Furthermore, deviant trajectories exhibited both accelerated (brain-age > PMA) and decelerated/delayed (brain-age <PMA) changes.

Finally, repeating the above analysis on comparatively simpler models that utilised QS only, non-QS only and the full-cycle periods, confirmed that combining both QS and non-QS was indeed the optimal choice. This additional study is presented in Appendix A.2.

### Biomarkers of maturation & neurodevelopmental outcome

**Figure 5:**
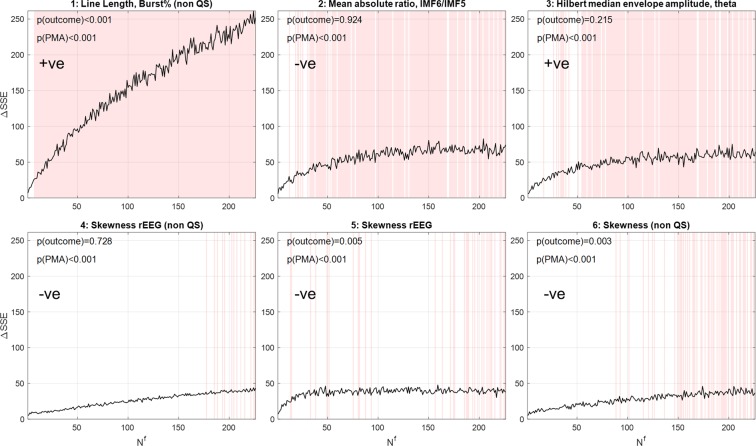
The top six features predominantly selected by the RF brain-age prediction models. Features are ranked according to the values of the $\Delta SSE$ at *N*^*f*^ = 226. $\Delta SSE$ is plotted for each feature across the full *N*^*f*^ range and shaded regions denote regions of *N*^*f*^ where the rank matched the rank at *N*^*f*^ = 226. The p-values for the PMA and outcome is also shown after a univariate regression of the individual feature data using linear mixed-effects models. ‘+ve’ and ‘−ve’ denote if the feature trends increased or decreased with PMA, respectively.

To identify which individual features were potential biomarkers of maturation and outcome, Fig. 5 shows the top six features that were prominently selected by the RF models. The feature ranking was decided from the $\Delta SSE$ values at the maximum ${N}^{f}$ = 226, and $\Delta SSE$ then plotted for the full *N*^*f*^ range. The shaded regions in each plot denote values of *N*^*f*^ where the feature rank matched the rank at *N*^*f*^ = 226. The p-values after fitting univariate regression models to each feature for PMA and outcome is also shown, along with a ‘+ve’ or ‘−ve’ to denote the direction of the feature’s trend with PMA.

The Line Length Burst% (in non-QS) showed by far the largest increase with *N*^*f*^ indicating that it was selected with increasing frequency by the model as a discriminating feature for predicting brain-age. It was the only feature that was consistently ranked as first across *N*^*f*^ and by showing a high individual significance for both PMA and outcome, was the driving feature behind the better separation of normal and abnormal outcome trajectories at high *N*^*f*^. Line Length Burst% measures the total percentage duration of burst EEG activity (which is strongly characteristic of preterm EEG) that occurs within a 30 second window, using the line length burst detection algorithm developed by *Koolen et al*.^23^, and showed a positive trend with PMA.

Mean absolute ratio of IM6/IMF5 also showed some prominence and the negative trend with PMA described the shift towards higher frequencies and less slow-wave activity in the EEG, while the positive trend in the Hilbert median envelope amplitude in theta described the increase in the amplitudes of the EEG within the theta (3–8 Hz) band (see Appendix A.1 for more detailed definitions). Skewness measures in QS and non-QS (reflecting the shift of the EEG amplitude distribution away from the normal distribution) were the next common features but were no longer consistently ranked. These findings show the dominance of the first three features, but most notably Line Length Burst% (in non-QS), and its efficacy as a maturational and outcome-sensitive biomarker.

**Figure 6:**
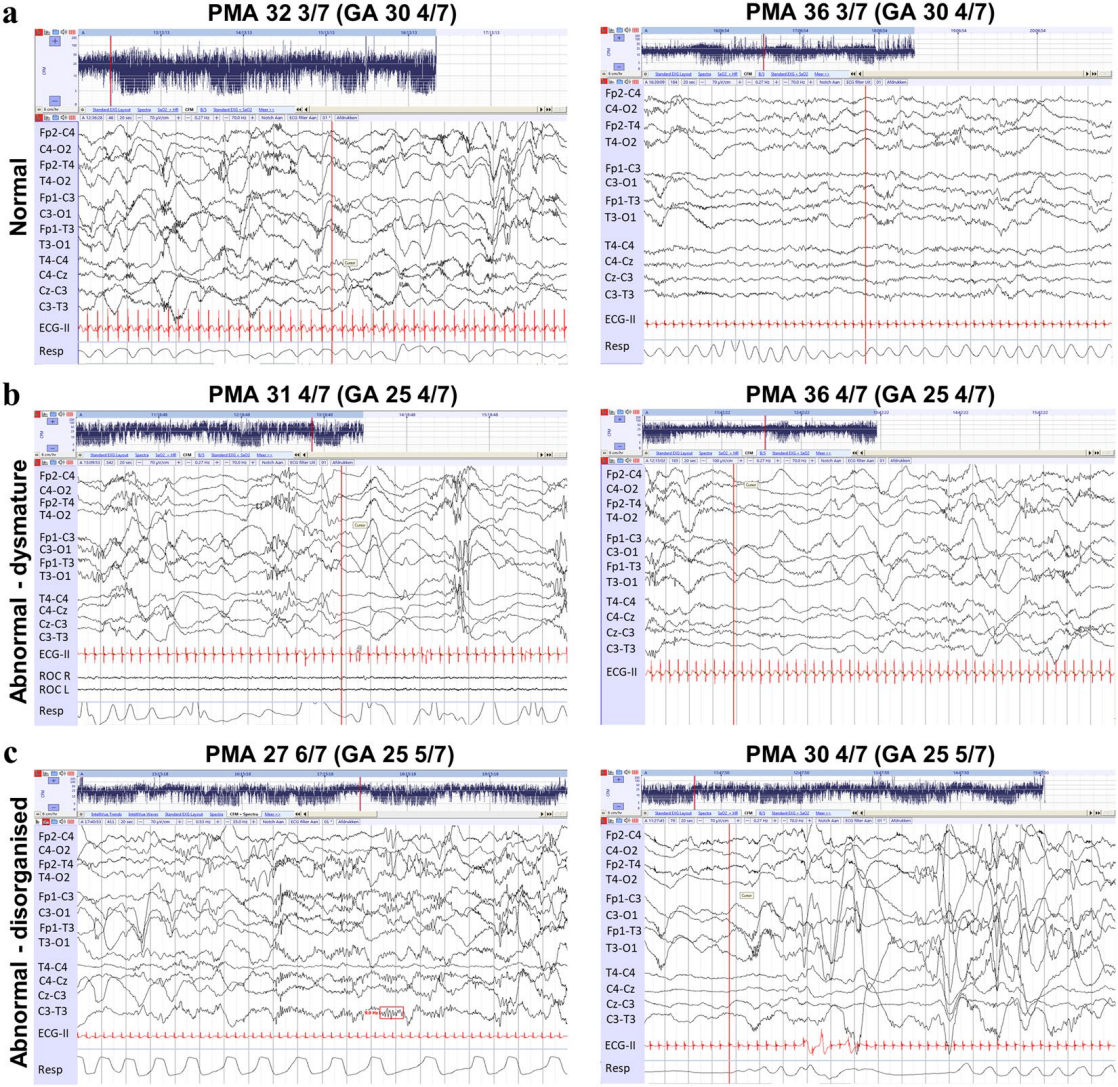
20 second non-QS EEG epochs extracted from recordings that formed a patient’s brain-age trajectory. On each EEG panel from top to bottom: First four EEG channels reflect the right hemisphere from anterior to posterior. Next four channels reflect left hemisphere from anterior to posterior. Channels 9–10 are right midline and channels 11–12 are left midline. The PMA and GA of the baby’s recordings (in weeks) and the overall clinical assessment of the EEG morphology (normal, dysmature or disorganised) are also given. (**a**) EEGs from a patient born at 30 4/7 weeks GA with a normal estimated brain-age trajectory and normal (clinically defined) EEG characteristics. (**b**) A patient born at 25 4/7 weeks GA with a delayed estimated brain-age trajectory and abnormal, dysmature EEG. (**c**) A patient born at 25 5/7. The first recording at 27 6/7 weeks PMA had an accelerated estimated brain-age trajectory and abnormal, disorganised EEG. The second recording at 30 4/7 weeks PMA had both disorganized and dysmature behaviour.

To better understand the behaviour of Line Length Burst% (non-QS) in relation to the EEG itself, Fig. 6 provides examples of recordings belonging to patients that presented normal and abnormal trajectories, focussing on non-QS EEG.

As the EEG matured normally (Fig. 6a), the significantly positive correlation of Line Length Burst% (in non-QS) and PMA (from Fig. 5) reflected the transition from clear discontinuous burst to inter-burst interval (periods of suppressed EEG between successive bursts) EEG behaviour of preterm babies, to a more continuous, less regular EEG activity towards term-age.

Resulting observed trajectory delays (as illustrated in Fig. 4) largely driven by Line Length Burst% (in non-QS) reflected changes in specific morphologies in the EEG, resulting in a delay for what was expected at the given age and a dysmature-labelled EEG (as shown in Fig. 6b).

Observed accelerated trajectories in this study were largely recording-specific and further inspection of the EEG recordings showed them to be predominantly disorganised (as opposed to dysmature), with one such recording shown in Fig. 6c. This was due to the EEG morphology exhibiting unusual deformed activity (e.g. ‘cogwheel-shaped’ brushes with high frequency components and repetitive rhythms), without signs of acute depressions (e.g. longer inter-burst intervals or periods of discontinuity appropriate for age) which is linked with abnormal neurodevelopmental outcome^43^. There were also abundant abnormal sharp transients, asymmetry, burst asynchrony and possible inter-ictal rhythmic activity^19,44,45^. This particular patient developed a severe brain lesion which was reflected through this abnormal evolution of their EEG behaviour.

## Discussion

This study utilises a data-driven approach to link early maturational deviations to neurodevelopmental outcome. It is the first method that successfully incorporates objective features as part of a brain-age model to achieve this task, over a wide PMA range, identifying key biomarkers of maturation and outcome. The approach also considered differences across sleep state and avoids the need for a ground truth at the time of recording.

Increasing *N*^*f*^ causes a rise in $RMSE$ and *δ*^*max*^ in those patients with abnormal neurodevelopmental outcome, while maintaining a consistently stable and excellent performance for normal outcome patients. Moreover, the majority of maturational deviations were driven by single-recording deviations in the trajectory rather than an overall change in trajectory correlation. Deviations also proved to exhibit both a decelerated and accelerated rate of maturation.

Trends in the behaviour with *N*^*f*^ is linked to the fundamental processes of the RF and further helps identify key biomarkers driving these changes. At low *N*^*f*^ values, few features are chosen randomly for selection for each split in a decision tree and this increases the range of features represented across the entire forest. The lack of significant differences between abnormal and normal outcomes at low *N*^*f*^ are due to the reduced specificity to outcome-sensitive features highlighting that, while many features show strong trends with PMA, not all can quantify the differences across outcome. This is one of the key limitations of existing brain-age models, which are validated only on normal outcome patients.

At high values of *N*^*f*^, the majority of the feature set is presented at each tree split increasing the chance of selecting the same feature multiple times. If there are features that are much more strongly correlated with PMA, this concentrates the model on this smaller subset of features. Here, we also observe the intriguing effect of improving the separation of outcome, as those features most strongly correlated to PMA also exhibit very strong outcome sensitivity.

More specifically, Line Length Burst% (in non-QS) provides the strongest correlations and is by far the most prevalent feature used in the brain-age models. It also proves to perform best within non-QS periods instead of the corresponding values for QS, which was previously used to demonstrate the efficacy of this feature^23^. This discovery motivates a more in-depth look into non-QS as a potentially more informative sleep state for assessing neurodevelopmental outcome.

By term age, subtle changes in the EEG background are apparent in QS by visual inspection (e.g. changes in inter-burst interval length and burst activity), and not solely due to the naturally reduced level of artefacts in this sleep state. However, the more severe the underlying functional disturbance, the more the EEG patterns will have changed over the whole sleep cycle (e.g. changes in asynchrony, amplitude and continuity) and hence also in non-QS^46^. During the preterm period, the well-described discontinuity (burst to inter-burst intervals) related to QS spans most of the preterm postnatal period before developing into continuous activity by term-age. On the other hand, active sleep (part of non-QS) shows an additional preceding period of semi-continuous activity. These additional transitionary phases during non-QS development may explain the better age discrimination of the Line Length Burst% in non-QS across PMA compared to other features in QS. It may also explain why a non-QS period of recording better highlights a deviating trajectory (see Fig. 6), as the corresponding discontinuity during QS is not yet sufficiently discriminative.

Linking delayed trajectory behaviour with the EEG identified a clear dysmaturity, while accelerated trajectories were most strongly linked with disorganised patterns. In the latter, we assume that such morphological changes to the EEG are reflected in the outcome-sensitive features and, consequently, it is more likely that the disorganised nature of the EEG activity results in key outcome-sensitive features exhibiting values not fitting with the age-specific values, but to a more ‘continuous’ EEG. This results in an over-estimation of the brain-age and accelerated trajectory that still correlates with abnormal outcome.

With these observations in mind, we note that delayed trajectories could also be caused by abnormal disorganised patterns (as well as delayed dysmaturity), such as due to an increased burst-suppression activity^47^. Similarly, accelerated trajectories may also correspond to an accelerated dysmature EEG (as well as disorganised activity). *Scher at al. (1997)* confirms the presence of accelerated dysmaturity in preterm development that still results in an abnormal outcome due to the increased speed of maturation^18^.

The presence of deviating trajectories could warrant extra EEG monitoring and/or brain imaging, as it may reflect altered developmentally regulated neuronal pathways^48,49^, which may be mediated by the effect of interventions/environmental stresses (e.g. deterioration after a sepsis episode, exposure to pain and extra-uterine environment, brain injury, episodes of cardiorespiratory instability etc.). Since babies had to be (respiratory and hemodynamically) stable during their EEG recordings, there was no intention to record EEG during acute adverse conditions. We did see changes in consecutive cranial brain ultrasound (see Table 1) that were more significant in the severe/death outcome group. We noticed that the EEG deviations frequently preceded persistent ultrasound brain abnormalities (if any) and therefore assume that the observed EEG changes reflect chronic stage abnormalities rather than acute stage abnormalities.

One of the limitations of this study remains that, while we introduce a serial analysis of EEG recordings per patient, there are only a limited number of recordings which are all measured at irregular age intervals. This is largely to maintain the delicate balance between accurate diagnostics and avoiding unnecessary disturbance to the vulnerable baby. It does, however, make it difficult to obtain a higher resolution timing of abnormal deviations. This said, *Le Bihannic et al. (2012)* and *Périvier et al. (2016)* showed in line with our results that transient moderate-to-severe EEG abnormalities (present on at least one EEG during consecutive measurements) based on visual inspection, were independent predictors of neurodevelopmental outcome^2,27^. *Hayashi-Kurahashi et al. (2012)* conducted serial EEG recordings during three time periods in preterm infants <34 weeks. EEG abnormality grades (based on visual assessment) on at least one EEG during the first month of life, was significantly correlated with neurodevelopmental outcome at 12 to 18 months^50^. Based on these study results, we hypothesize that one abnormal EEG recording may already reflect impaired neuronal pathways, and this becomes more prominent when performing a serial recording study^50,51^.

It is difficult to quantitatively compare our data with other maturational studies since these did not include abnormal neurodevelopmental outcome in the same manner. This said, *Tokariev et al. (2019*) did find, based on quantitative analysis of one EEG at term equivalent age in extremely preterm infants (around 26 weeks GA), a difference in sleep-state-related connectivity in the posterior networks. This was significantly correlated with visual performance and showed a non-significant negative correlation with social-emotional performance at 24 months follow-up^52^.

Neurodevelopmental assessment at nine months of corrected age in one of the cohorts (i.e. ${\mathcal{D}}2$) is a second limitation of this study and may underestimate more subtle motor or behavioural disturbances which may only become apparent at later follow-up. Furthermore, separating into outcome groups results in reduced data sizes, inhibiting robust pre-selection of the outcome-sensitive features before performing brain-age prediction. These limitations make it challenging to derive a precise and reliable threshold for identifying deviating trajectories, preventing the model from performing a fully automated classification of outcome at this point in time.

27 female and 38 male preterm infants were analysed in total, with significantly more preterm males in the abnormal outcome group. Male disadvantage in the preterm population has been a consistent finding over the past decades, including higher rates of preterm birth, increased mortality and (long-term) morbidity^53,54^. Clarifying the underlying mechanism of gender-unequal consequences of prematurity is an important topic as it might become a target for precision medicine. We have previously tested on ${\mathcal{D}}1$ (normal outcome) the effect of gender on the maturation of some of the features defined in this study (including Line Length Burst%) and did not find a significant gender effect in this group with normal neurodevelopmental outcome^55^. *Griesmaier et al. (2014)* did find sex-related differences in the maturation of amplitude-integrated EEG signals during the first four weeks, however, these results were not correlated with outcome^56^. In future studies with larger data sizes, it would be of major interest to delineate whether sex-related differences in maturational trajectories can be observed and whether these altered neurophysiological mechanisms can be related to long-term outcomes.

We also acknowledge the important effect of commonly used drugs (such as fentanyl, morphine, caffeine and phenobarbital) in the NICU on early brain activity^12^. The complex interplay between optimal comfort/pain management and exposure to pain-relieving sedative drugs (all with potential adverse long-term consequences^57,58^) challenges current neonatal practice for compassionate treatment. Assessing the effect of both sedatives and pain on functional brain maturation is a complex but intriguing question. Since these premature infants were mainly recorded after the first week of life, only one baby received sedative drugs (Fentanyl) during the recording. Fentanyl can suppress the EEG and lengthen the inter-burst intervals which could theoretically influence Line Length Burst% and other features. *Malk et al. (2014)* showed how EEG bursts were also influenced by drugs (changes in duration and enhanced oscillation at higher frequencies) and thus presented altered EEG characteristics^12^.

All babies received caffeine until 35–36 weeks PMA. Caffeine has been reported to influence EEG continuity, so the effect is considered to be applicable to all. In-depth drug analysis is beyond the scope of this paper but is certainly an important direction to explore if we wish to achieve future bedside implementation of these monitoring techniques.

In summary, future work requires a more regular (ideally weekly) recording of EEG and a larger data size to train models specifically on outcome-sensitive features, additionally including corrections for gender and drug use. Increasing data size and resolution may produce more detailed trajectories that may better show subtler changes in behaviour due to altered neuronal pathways. It could also open the possibility of directly and reliably classifying the deviations as normal or abnormal, by introducing robust thresholds based on the magnitude of the trajectory deviations and/or latency. From this, the relationship between outcome estimation and number of EEGs recorded could also be investigated. Efforts are being made within the neonatal research community to better share and expand such databases.

This important study cements the link between brain-age trajectories and neurodevelopmental outcome. Furthermore, by presenting an approach that identifies the important sleep states, combines an objective set of data-driven features (of which key outcome-sensitive maturational biomarkers are identified) and avoids the need for visual grading at the recording level, it is an important next step towards the automated early identification of abnormal neurodevelopmental outcome.

## Appendix

### A.1 Feature extraction

As listed in Table 2, the data-driven feature set included amplitude-based features such as skewness (a measure of similarity of the amplitude distribution compared to a normal distribution, where negative values denote the a left-sided skew, and positive values a right-sided skew), as well as measures of signal complexity such as Multiscale Entropy (MSE)^35^ and fractal dimension.

The Line Length method of *Koolen et al*.^23^ measures changes in the amplitude and frequency of the signal across successive one-second windows, using a threshold to then classify high amplitude, high frequency periods as EEG bursts. The percentage of the total duration that are burst periods within a 30 second window is then defined as the Line Length Burst%.

The rEEG^24^ similarly summarizes discontinuity by identifying the inter-burst intervals in the signal after converting the multichannel EEG to an approximation of amplitude-integrated EEG.

Features were also derived from EEG decompositions. Classical delta (0.5–3 Hz), theta (3–8 Hz), alpha (8–12 Hz) and beta (12–30 Hz) band powers were determined from the Fourier transform of the signal. Related decompositions based on the Discrete Wavelet Transform (DWT)^59^ and Empirical Mode Decomposition (EMD)^60^ were also calculated to better capture the signal’s non-stationarity. The DWT decomposed the signal into Detail and Approximation bands up to the fifth level (denoted D3 to D5 and A5, respectively)^59^ while the EMD produced six bands known as Intrinsic Mode Functions (denoted by the set IMF1-IMF6)^60^. IMF1 contains the highest frequency information and IMF6 the lowest frequencies. Variation coefficient of each IMF provided a mean-standardized measure of the signal variance and fluctuation index measured the intensity of the signal change.

Further applying the Hilbert transform to the IMFs and Fourier bands obtained band-specific measures of signal envelope and instantaneous frequency^61,62^.

### A.2 Comparison of results across sleep states

When comparing the models for QS, non-QS, combining QS and non-QS, and the full-cycle periods, all models were analysed in the same manner as for the combined QS and non-QS model presented in the main results. The stable range for each model was observed to be always from *N*^*f*^ = 20 to the maximum *N*^*f*^ value (113 for QS only, non-QS only and full-cycle models, and 226 when combining QS and non-QS). Results are presented in Table 3. Asterisks in the column headings denote that the values were part of a statistically significant cluster (p < 0.05) across *N*^*f*^. The maximum median differences between normal and abnormal groups within the significant cluster (or across the stable range if there was no significance) were calculated for each trajectory summary metric, as well as the median values at those locations for both normal and abnormal outcomes. As a further comparison, Cliff’s delta was also determined at these locations as a measure of effect size, quantifying the degree of overlap between the normal and abnormal groups^63^ (values > 0.28 denote a medium to large effect size). Comparisons using $r$ were not included here as it proved uninformative based on the results in the main body of text.

**Table 3:** Comparison of the trajectory summary metrics for models using features from only QS, only non-QS, QS and non-QS, and non-specific two-hour periods of EEG containing at least one full sleep cycle.

		QS*	non-QS*	QS & non-QS*	full-cycle
*RMSE* (weeks)	normal	0.85 (0.61–1.45)	0.86 (0.69–1.23)	0.76 (0.62–1.43)	0.75 (0.56–1.32)
	abnormal	1.36 (1.00–1.79)	1.40 (1.08–1.84)	1.36 (1.17–1.55)	1.23 (0.90–1.43)
	**difference**	**0.51**	**0.54**	**0.61**	**0.48**
	**Cliff’s Delta**	**0.38**	**0.43**	**0.41**	**0.32**
*δ*^*max*^ (weeks)	normal	1.00 (0.75–1.91)	1.14 (0.89–1.88)	0.93 (0.78–1.64)	1.05 (0.74–1.79)
	abnormal	1.90 (0.87–2.75)	2.01 (1.44–3.15)	1.80 (1.20–2.85)	1.69 (0.98–2.43)
	**difference**	**0.90**	**0.87**	**0.87**	**0.64**
	**Cliff’s Delta**	**0.21**	**0.39**	**0.40**	**0.30**

Values for the normal and abnormal outcome trajectories are both given, with medians and the interquartile ranges (in brackets) provided (where appropriate). Differences between these values are also given, along with the Cliff’s delta. The results are presented at values of *N*^*f*^ within each significant cluster where the differences were maximal. Asterisks in the column headings denote that the results were part of a statistically significant cluster.

From the results in Table 3, all models had a statistically significant cluster, apart from the full-cycle periods, showing the importance of separating for sleep state. While combined QS & non-QS and non-QS only models showed better values for the median difference and Cliff’s delta (compared to QS only), QS & non-QS had a better RMSE and *δ*^*max*^ in the normal outcome group than non-QS only. Overall, this showed that the more complex model which combined both QS and non-QS was indeed the most suitable choice.

## Data Availability

The research materials and data supporting this publication can be accessed, upon reasonable request, by contacting the corresponding author.
